# Human Cardiac Mesenchymal Stromal Cells with CD105^+^CD34^-^ Phenotype Enhance the Function of Post-Infarction Heart in Mice

**DOI:** 10.1371/journal.pone.0158745

**Published:** 2016-07-14

**Authors:** Justyna Czapla, Sybilla Matuszczak, Ewa Wiśniewska, Magdalena Jarosz-Biej, Ryszard Smolarczyk, Tomasz Cichoń, Magdalena Głowala-Kosińska, Joanna Śliwka, Marcin Garbacz, Mateusz Szczypior, Tomasz Jaźwiec, Agnieszka Langrzyk, Michał Zembala, Stanisław Szala

**Affiliations:** 1 Center for Translational Research and Molecular Biology of Cancer, Maria Skłodowska-Curie Memorial Cancer Center and Institute of Oncology, Gliwice Branch, Gliwice, Poland; 2 Department of Cardiac Surgery and Transplantology, Silesian Center for Heart Diseases, Zabrze, Poland; National Cancer Institute, UNITED STATES

## Abstract

**Aims:**

The aim of the present study was to isolate mesenchymal stromal cells (MSC) with CD105^+^CD34^-^ phenotype from human hearts, and to investigate their therapeutic potential in a mouse model of hindlimb ischemia and myocardial infarction (MI). The study aimed also to investigate the feasibility of xenogeneic MSCs implantation.

**Methods and Results:**

MSC isolated from human hearts were multipotent cells. Separation of MSC with CD105^+^CD34^-^ phenotype limited the heterogeneity of the originally isolated cell population. MSC secreted a number of anti-inflammatory and proangiogenic cytokines (mainly IL-6, IL-8, and GRO). Human MSC were transplanted into C57Bl/6NCrl mice. Using the mouse model of hindlimb ischemia it was shown that human MSC treated mice demonstrated a higher capillary density 14 days after injury. It was also presented that MSC administrated into the ischemic muscle facilitated fast wound healing (functional recovery by ischemic limb). MSC transplanted into an infarcted myocardium reduced the post-infarction scar, fibrosis, and increased the number of blood vessels both in the border area, and within the post-infarction scar. The improvement of left ventricular ejection fraction was also observed.

**Conclusion:**

In two murine models (hindlimb ischemia and MI) we did not observe the xenotransplant rejection. Indeed, we have shown that human cardiac mesenchymal stromal cells with CD105^+^CD34^-^ phenotype exhibit therapeutic potential. It seems that M2 macrophages are essential for healing and repair of the post-infarcted heart.

## 1. Introduction

In late 1970s the Friedenstein group first isolated and described the basic features of mesenchymal cells present in the bone marrow of rodents [1–4].

Until now, mesenchymal stromal cells (MSC) are the subject of many studies worldwide. The existing data suggest that MSC can participate in a variety of therapeutic processes [5,6]. MSC may be involved in: (1) bone, cartilage, and muscle regeneration [7]; (2) improvement of the function of the post-infarction heart [8], regeneration of damaged liver and wound healing [9]; (3) inhibition of abnormal immune response [10]; (4) or may serve as carriers of therapeutic genes [11].

MSC are attributed to four primary mechanisms of action [12,13]: (1) MSC are able to home to the inflammatory areas; (2) MSC are able to transdifferentiate into, for example, cardiomyocytes, endothelial cells, smooth muscles cells; (3) MSC secrete factors that stimulate regeneration and repair of damaged tissues and organs; (4) MSC possess immunomodulatory properties (they inhibit, among others, inflammation). For this reason, MSC are called „guardians of inflammation” [14].

The aim of our study was to find an answer to questions: Do human MSC with CD105^+^CD34^-^ phenotype possess therapeutic properties, in other words, do they facilitate the functional recovery of ischemic hindlimb and infarcted mouse heart? Do therapeutic properties of MSC may be tested in xenogeneic system?

In the study we investigated therapeutic potential of MSC with CD105^+^CD34^-^ phenotype. The choice to use these cells may be explained by four reasons. (1) CD105 antigen (endoglin, TGFβ coreceptor) is a characteristic marker for mesenchymal cells [15]. (2) MSC selected for the presence of CD105 exhibited significantly better therapeutic properties than unselected cells [16]. Endoglin activates pathways involved in scar remodeling, and protects endothelial cells against apoptosis induced by hypoxia [17]. (3) A purified CD105^+^ cells population has a reduced heterogeneity, which may influence its potency, safety, tissue specific efficacy and mechanism of action [18]. (4) It has also been proven that cells isolated from hearts have a greater therapeutic potential than cells isolated from bone marrow [19].

Our data confirm therapeutic properties of human MSC with CD105^+^CD34^-^ phenotype. Human MSC stimulate angiogenesis in mouse ischemic tissues, reduce the post-infarction scar and fibrosis, and increase the left ventricular ejection fraction in a model of murine MI. Moreover, we did not observe MSC rejection. Additionally, MSC secrete factors which may polarize macrophages toward anti-inflammatory M2 phenotype [20]. It seems that M2 macrophages are essential for healing and repair of the post-infarcted heart [21,22].

## 2. Materials and Methods

An expanded Methods section can be found in the supplementary material online (S1 Text).

### 2.1. Ethical statement

The experiments were performed in accordance with the Declaration of Helsinki, with the approval of the Local Committee on Bioethics in Katowice. All patients provided written informed consent for the collection of excised heart and subsequent analysis.

Mice (6- to 8-week-old, C57Bl/6NCrl males) were bred in our animal facility house. The experimental protocol was approved by the Local Ethics Commission (Medical University of Silesia, Katowice, Poland).

### 2.2. Isolation of CD105^+^CD34^-^ cells

The tissue collected from human hearts was minced into fragments of approximately 1mm^3^ and digested with collagenase using a modification of as previously described protocol [19].

After obtaining 90–100% confluence of MSC, the cells were incubated with antibodies directed against human antigens: CD105 and CD34. The population of CD105^+^CD34^-^ cells was separated using a BD FACSAria™ III cell sorter (BD Biosciences). The cells from the 1^st^ to 3^rd^ passages were used for further experiments.

### 2.3. Immunophenotypic analysis and differentiation of MSC with CD105^+^CD34^-^ phenotype

The phenotype of CD105^+^CD34^-^ cells (1st passage) was determined using a flow cytometer (BD FACSCanto ™ BD Biosciences). The cells were incubated with appropriate antibodies directed against the following human antigens: CD29 –FITC (eBioscience), CD105 –APC, CD73 –PE, CD90 –PE-Cy7, CD44 –FITC, CD34 –PE-Cy7, CD31 –FITC, CD146 –PE, KDR–PE, LIN–FITC, CD45 –PE, HLA-DR–PE-Cy7 (BD Pharmingen), or isotype-matched control antibodies were used. The Human Mesenchymal Stem Cell Functional Identification Kit (R&D Systems, Minneapolis, MN, USA) was used to differentiate CD105^+^CD34^-^ cells into adipocytes, osteoblasts, and chondroblasts. The procedure was performed in accordance with the manufacturer’s instructions.

### 2.4. Analysis of the secretome of CD105^+^CD34^-^ cells

The type and quantity of cytokines and growth factors secreted by CD105^+^CD34^-^ cells, was assessed using the Human Cytokine Antibody Array C5 kit (RayBiotech, Norcross, GA, USA). The analysis was conducted in accordance with the manufacturer’s instructions.

### 2.5. Mouse model of hindlimb ischemia

Unilateral femoral artery ligation was performed as described [23]. An hour after ligation, 10^6^ [23] of CD105^+^CD34^-^ cells in 100μL of PBS^-^ or 100μL of PBS^-^ were administered into the C57Bl/6NCrl male mice muscle. The function of the limb were assessed at the following time points: 2, 5, 7, 9, 12 and 14 days after artery ligation. Limb function were assessed on a scale of 0–3 points according to the following criteria [24]: 3 –most severe, unable to use the foot; 2 –no dragging, ability to flex the angle, able to use the limb as weight support; 1 –positive plantar flexion, some problems can be observed as compared to the healthy limb; 0 –no difference between the ligated limb and the healthy (control) limb can be observed.

### 2.6. Induction of myocardial infarction (MI) and CD105^+^CD34^-^ cells implantation

MI was induced by ligation of the left anterior descending coronary artery (LAD) in 8-10-week-old C57Bl/6NCrl male mice kept under anesthesia. 7 days after LAD ligation, second thoracotomy was performed and 0.5x10^6^ CD105^+^CD34^-^ cells suspended in 10μl of PBS^-^ or 10μl of PBS^-^ (control), were injected into the border area of the post-infarction scar. The sham group consisted of mice injected with PBS^-^ without LAD ligation. Hearts were collected 42 days after PBS^-^ or CD105^+^CD34^-^ cells injection [16].

### 2.7. Immunohistochemistry

Formalin-fixed heart were embedded in paraffin or frozen in liquid nitrogen and sectioned into 5–8μm slices. Viable tissue and collagen were visualized by incubation with a 0.1% solution of Fast Green and a 0.1% solution of Sirius Red (Sigma) in 1.2% picric acid (1h, RT). Blood vessels were detected by incubation with either lectin conjugated with FITC (*Lycopersicon Esculentum* Lectin LEL, Vector Laboratories) or CD31 (Abcam) antibody and FITC-conjugated secondary antibody. Macrophages were stained with appropriate antibodies (CD206 or iNOS; Abcam) and fluorochrome-conjugated secondary antibodies. Human cells in a mouse tissue were identified by human lamin A+C, Nuclear Envelope Marker (Abcam). IL -6 was stained with anti-IL-6 antibody (Abcam) and Texas Red-conjugated secondary antibody. Nuclei were counterstained by DAPI.

### 2.8. Identification of M1 and M2 macrophages

Flow cytometry was used to determine macrophages subset in the post-infarcted hearts. At the following time points: 1, 3 and 7 days, hearts were collected, minced and digested with collagenase solution.

### 2.9. Immunophenotypic analysis of BMDM

BMDM (Bone Marrow-Derived Macrophages) were isolated and differentiated as previously described [25,26]. Seven days after isolation, BMDM were incubated for 48h with conditioned medium from CD105^+^CD34^-^ cells (MSC-CM), control medium (IMDM medium supplemented with 20% FBS, L-glutamine and antibiotics) or control medium with lipopolysaccharide (LPS; 1μg/mL [27,28], eBioscience). Flow cytometry were used to determine the phenotype of BMDM from each group.

### 2.10 Statistics

Data were presented as mean ± standard deviation and compared between groups using Student’s t-test, or by one-way analysis of variance (ANOVA), using Tukeys’ multiple comparison test for post hoc analysis, with p < 0.05 considered statistically significant. Non-parametric testing (Kruskal-Wallis test, U Mann Whitney test) were used in the case of data that do not have a normal distribution. Statistical analysis was performed using Statistica 10 software.

## 3. Results

### 3.1. MSC isolated from the heart

**Fig 1:**
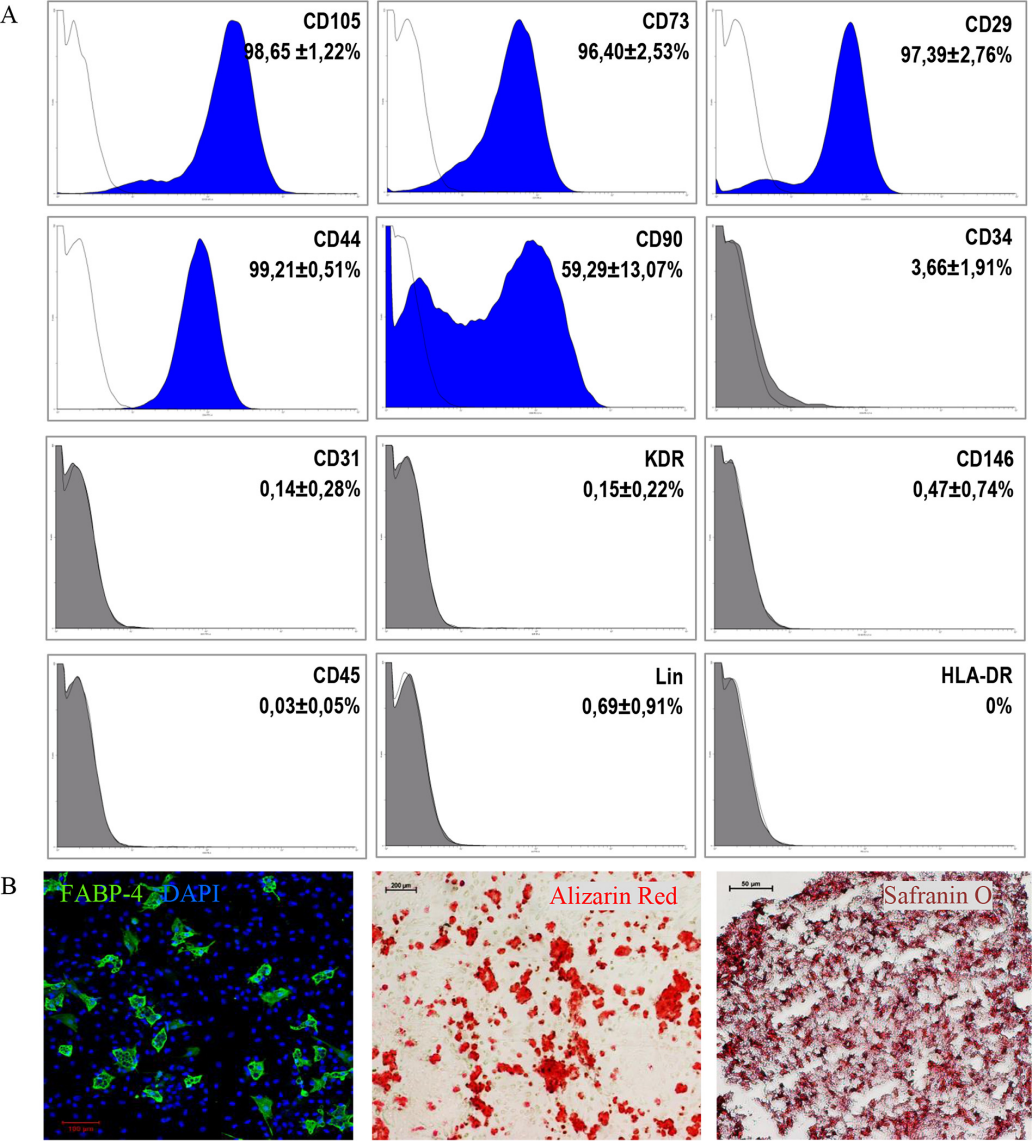
**Phenotype and differentiation potential of CD105**^**+**^**CD34**^**-**^ **cells selected from the MSC population** (A) Phenotype and differentiation potential of CD105^+^CD34^-^ cells selected from the MSC population isolated from fragments of heart tissue. Over 96% of the cells express markers characteristic for mesenchymal cells: CD105, CD29, CD73, and CD44, with the exception of the CD90 marker (~59.3%) (n = 11). CD105^+^CD34^-^ cells in culture do not express the following antigens (<1%): CD45, Lin, HLA-DR, CD31, KDR, and CD146. Despite the selection of the CD105^+^CD34^-^ population with high specificity of the process (P = 97.68±1.52%, n = 11), the presence of CD105^+^CD34^+^ cells (~3.7%) was still observed in the culture. (B) The cells *in vitro* differentiate into adipocytes (FABP4, green, n = 6, magn. 10x), osteoblasts (Alizarin Red, red, n = 6, magn. 4x), and chondroblasts (Safranin O, dark red, n = 4, magn. 20x).

The culture of MSC was established for 19 tissue fragments derived from the right ventricle. After obtaining 90–100% MSC confluence, a population of CD105^+^CD34^-^ cells was sorted. Cell surface antigen phenotyping of CD105^+^CD34^-^ cells (1st passage) indicated that over 96% of the cells expressed markers characteristic for mesenchymal cells, such as CD105, CD29, CD73, and CD44 (Fig 1A). The exception was the CD90 marker, whose percentage of cells equaled on average 59.3%. CD105^+^CD34^-^ cells in culture were devoid of (<1%) hematopoietic marker (CD45) and mature blood cells markers (LIN), and they did not express molecules of the major histocompatibility complex class II (HLA-DR). Among CD105^+^CD34^-^ cells there were no cells expressing a marker specific for mature endothelial cells (CD31), progenitor cells of the vessels (KDR), or CD146^+^ cells. We have shown previously that MSC isolated from human adult hearts lack c-Kit antigen [29]. CD105^+^CD34^-^ cells were able to differentiate *in vitro* into adipocytes, osteoblasts, and chondroblasts (Fig 1B).

### 3.2. Secretome of CD105^+^CD34^-^ cells

**Fig 2:**
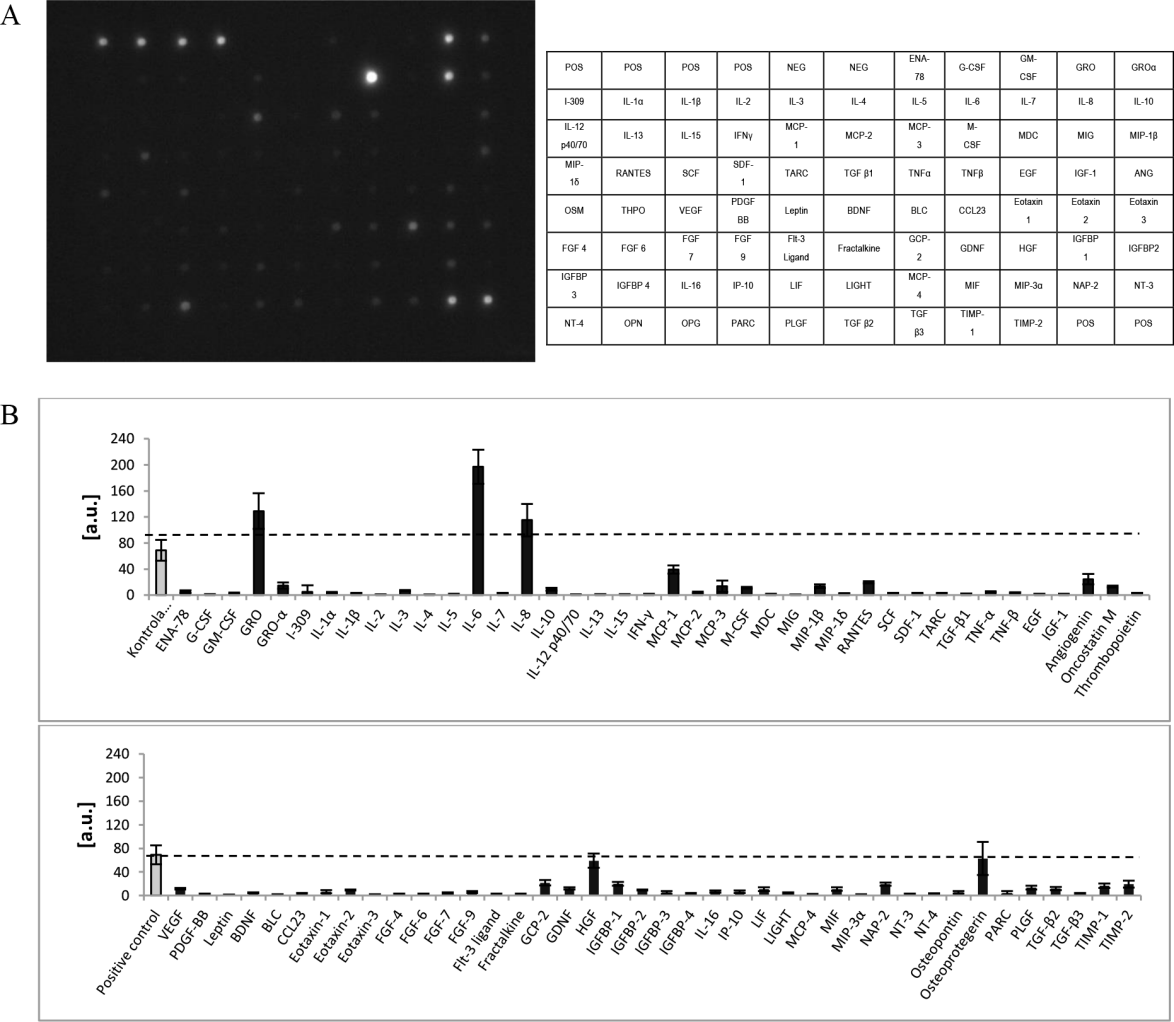
Cytokines and growth factors secreted by CD105^+^CD34^-^ cells *in vitro*. (A) An exemplary image of a membrane used for the analysis of 80 cytokines and growth factors secreted by CD105^+^CD34^-^ cells. (B) Densitometric analysis of 80 cytokines and growth factors secreted by CD105^+^CD34^-^ cells indicates that CD105^+^CD34^-^ cells secrete mainly IL-6, IL-8, and GRO molecules (n = 5).

Over 48h CD105^+^CD34^-^ cells in a cell culture secrete into the medium predominantly interleukin 6 and 8, as well as GRO molecules (chemokine C-X-C motif ligands) (Fig 2). In comparison with all analyzed cytokines, the cells also secrete a greater amounts of HGF and soluble molecules of the OPG (osteoprotegerin) receptor. IL-6 is the predominant cytokine secreted by the examined cells.

### 3.3. Mouse model of hindlimb ischemia

**Fig 3:**
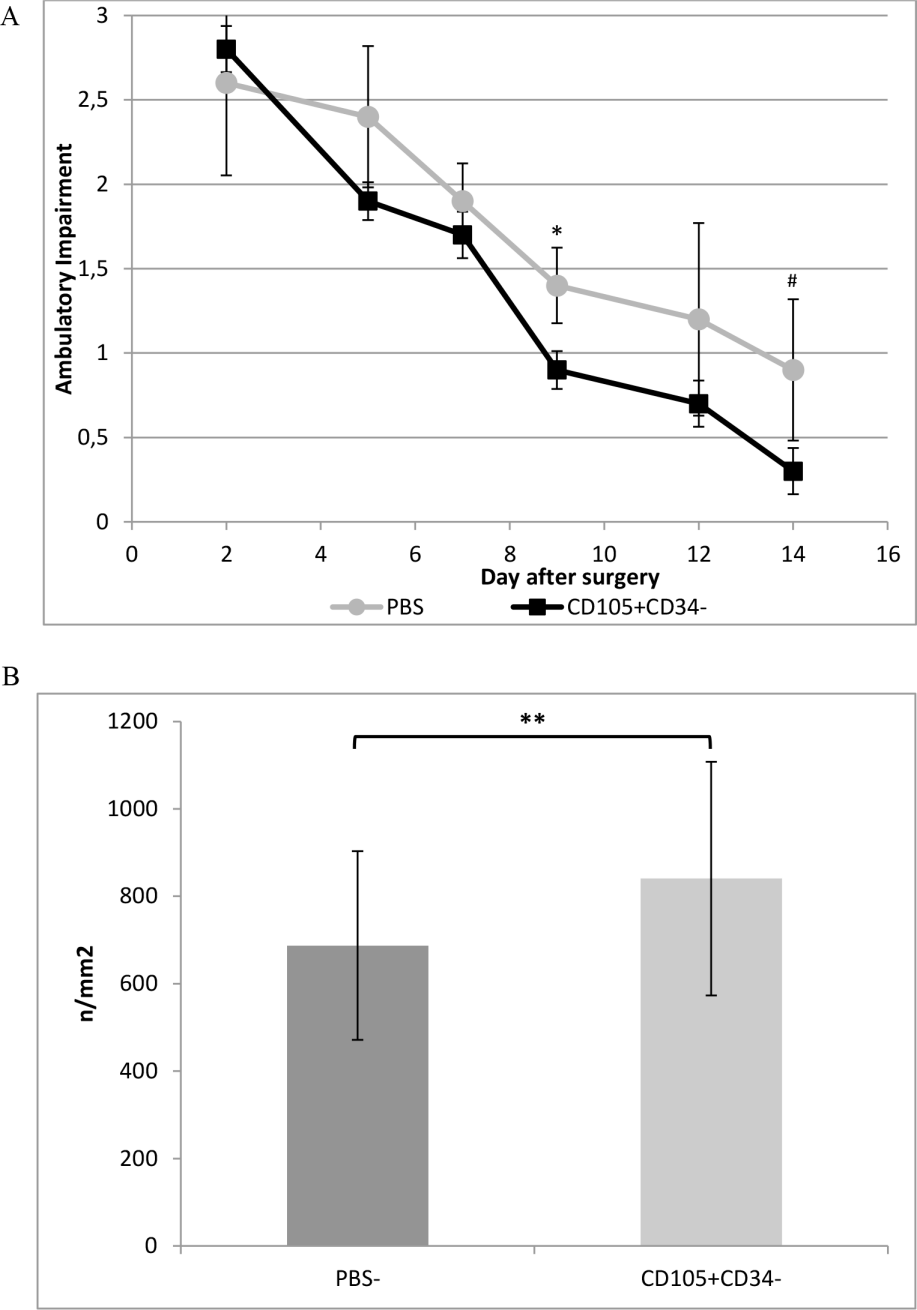
Therapeutic potential of CD105^+^CD34^-^ cells tested in a mouse model of hindlimb ischemia. (A) CD105^+^CD34^-^ cells treated mice demonstrated improved functional outcomes compared to the control mice (PBS^-^) (n = 5; experiment repeated twice). (B) The number of blood vessels was higher at day 14 in mice after administration of CD105^+^CD34^-^ cells compared to the control mice after administration of PBS^-^ (n = 10; 10 muscles per group were analyzed, in each muscle 10 pictures were taken). *p<0.05 #p = 0.056 **p<0.01 by the Mann-Whitney U test.

Mouse model of hindlimb ischemia allowed to assess proangiogenic features of CD105^+^CD34^-^ cells. Injection of one million CD105^+^CD34^-^ cells into the ischemic muscle facilitated fast wound healing (functional recovery by ischemic limb) (Fig 3A). Such an effect were not observed in the control group (mice that received PBS^-^). After 28 days, the mice in both groups are recovering the full functionality in ischemic limb [23]. After injection of CD105^+^CD34^-^ cells into ischemic muscle the increase in the number of blood vessels (840±247.1/mm^2^) in comparison with the control group (687.3±215.8/mm^2^) was observed (p<0.01) (Fig 3B).

### 3.4. CD105^+^CD34^-^ cells affect the increase in the number of blood vessels after MI

**Fig 4:**
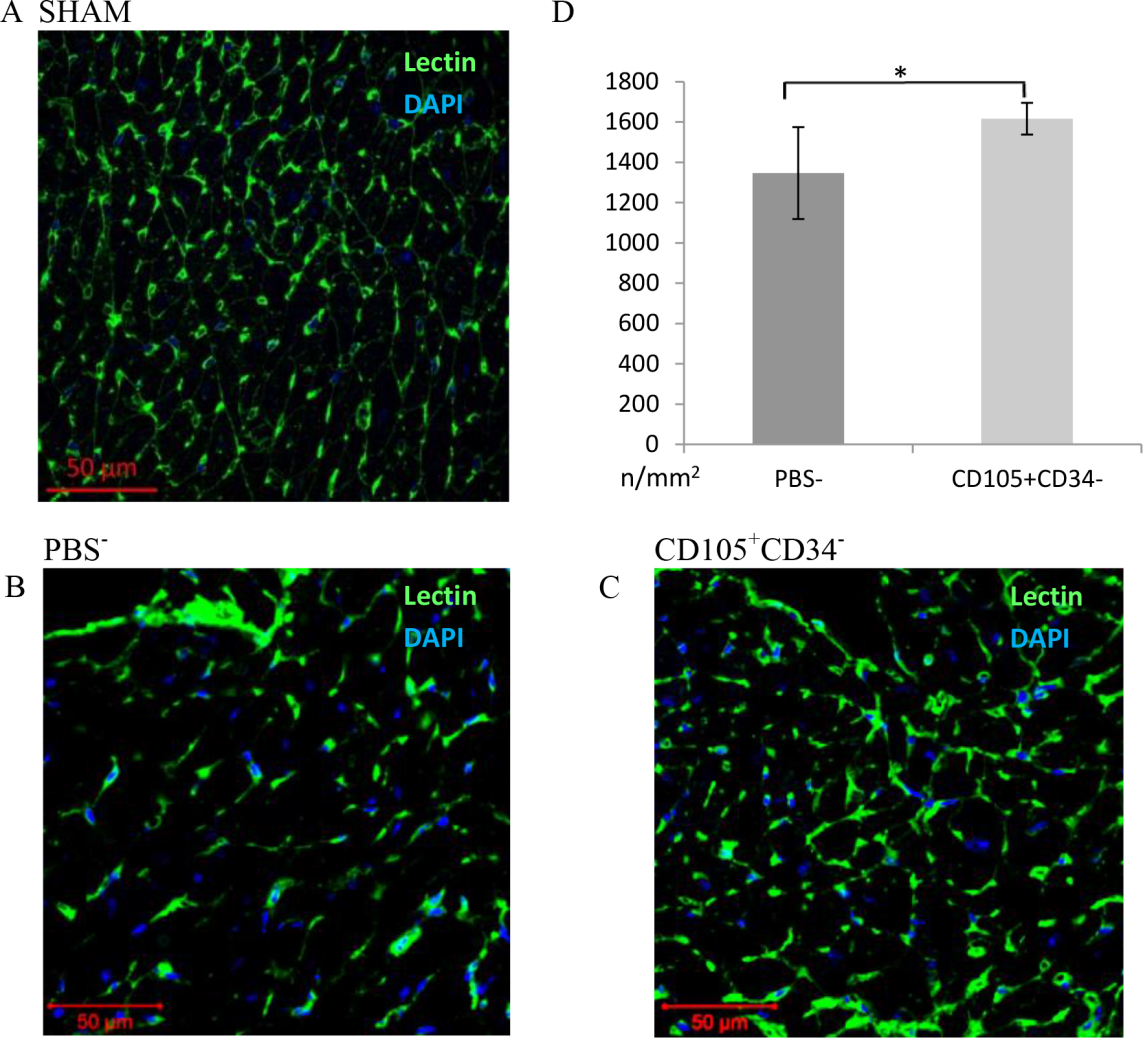
Capillary density in the border area of the post-infarction scar 7 weeks after MI. (A B C) Representative images presenting the number of blood vessels in the border area of the post-infarction scar (magn. 20x) (A) in a Sham group, (B) in mice after administration of PBS^-^, (C) in mice after administration of CD105^+^CD34^-^ cells. (D) In the area bordering the post-infarction scar a significant increase in the number of blood vessels in mice after administration of CD105^+^CD34^-^ cells compared to the control mice after administration of PBS^-^ was observed. n = 7; * p<0.05 by the Student’s t-test.

**Fig 5:**
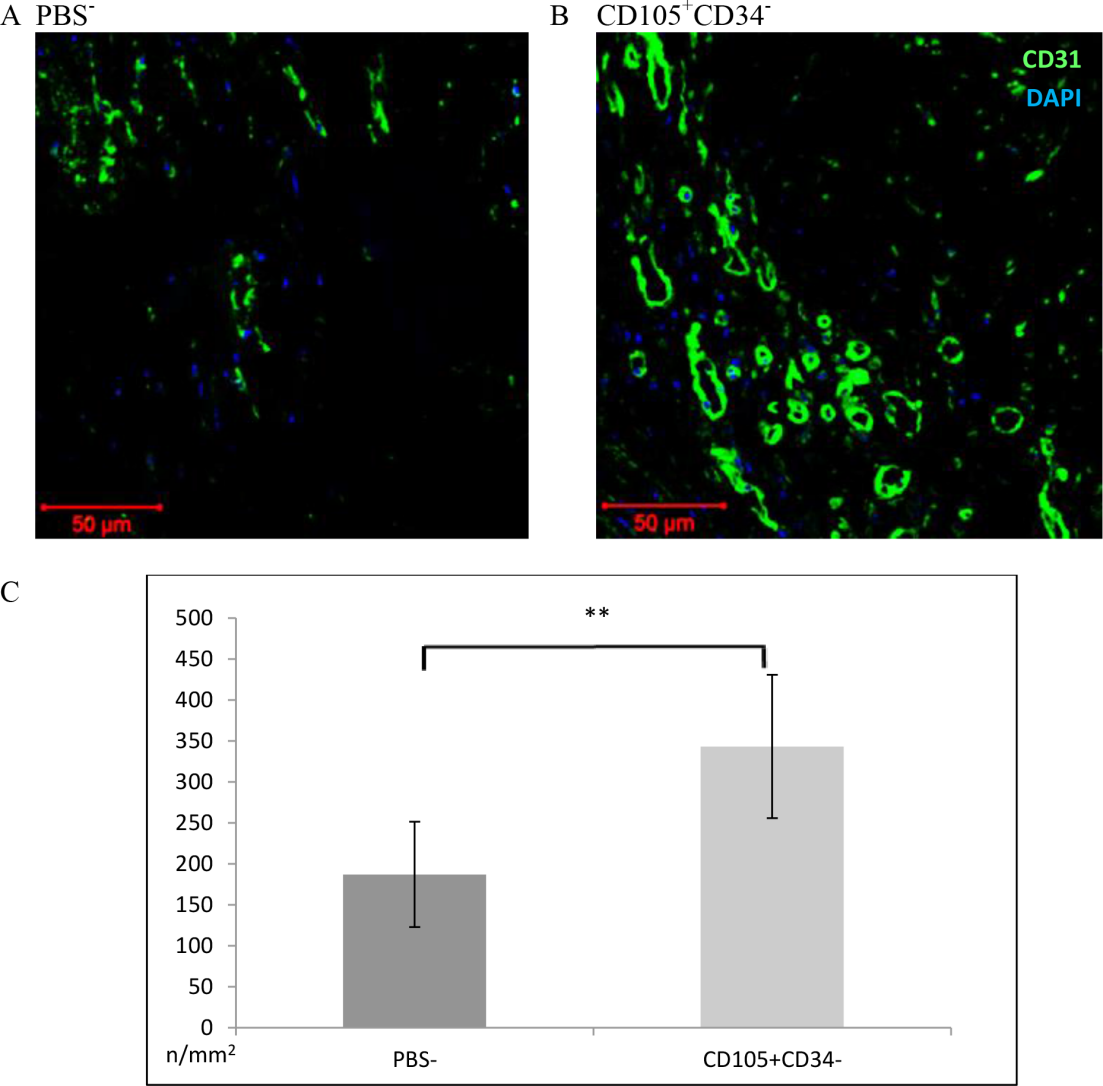
Capillary density within the area of the post-infarction scar 7 weeks after MI. (A B C) Representative images presenting the number of blood vessels within the area of the post-infarction scar. Magn. 20x. (A) in mice after administration of PBS^-^ (B) in mice after administration of CD105^+^CD34^-^ cells. (C) Within the scar, a significant increase in the number of blood vessels in mice after administration of CD105^+^CD34^-^ cells compared to the control mice after administration of PBS^-^ was observed. n = 7; ** p<0.01 by the Student’s t-test.

The assessment of the number of blood vessels was performed 6 weeks after MI. In the border area of the post-infarction scar, the vessels were stained with lectin (Fig 4). After 6 weeks a statistically significant increase in the density of capillaries in mice after administration of CD105^+^CD34^-^ cells (1616±39/mm^2^) compared to the control group (mice that received PBS^-^) (1347±114/mm^2^) (p<0.05) was observed (Fig 4D). The number of vessels located within the area of the post-infarction scar was determined using anti-CD31 antibody (Fig 5). A statistically significant increase in the number of vessels in mice after administration of CD105^+^CD34^-^ cells (343±44/mm^2^) compared to the control group (178±32/mm^2^) (p<0.01) was observed (Fig 5C).

### 3.5. CD105^+^CD34^-^ cells influence the reduction in the size of the post-infarction scar and fibrosis

**Fig 6:**
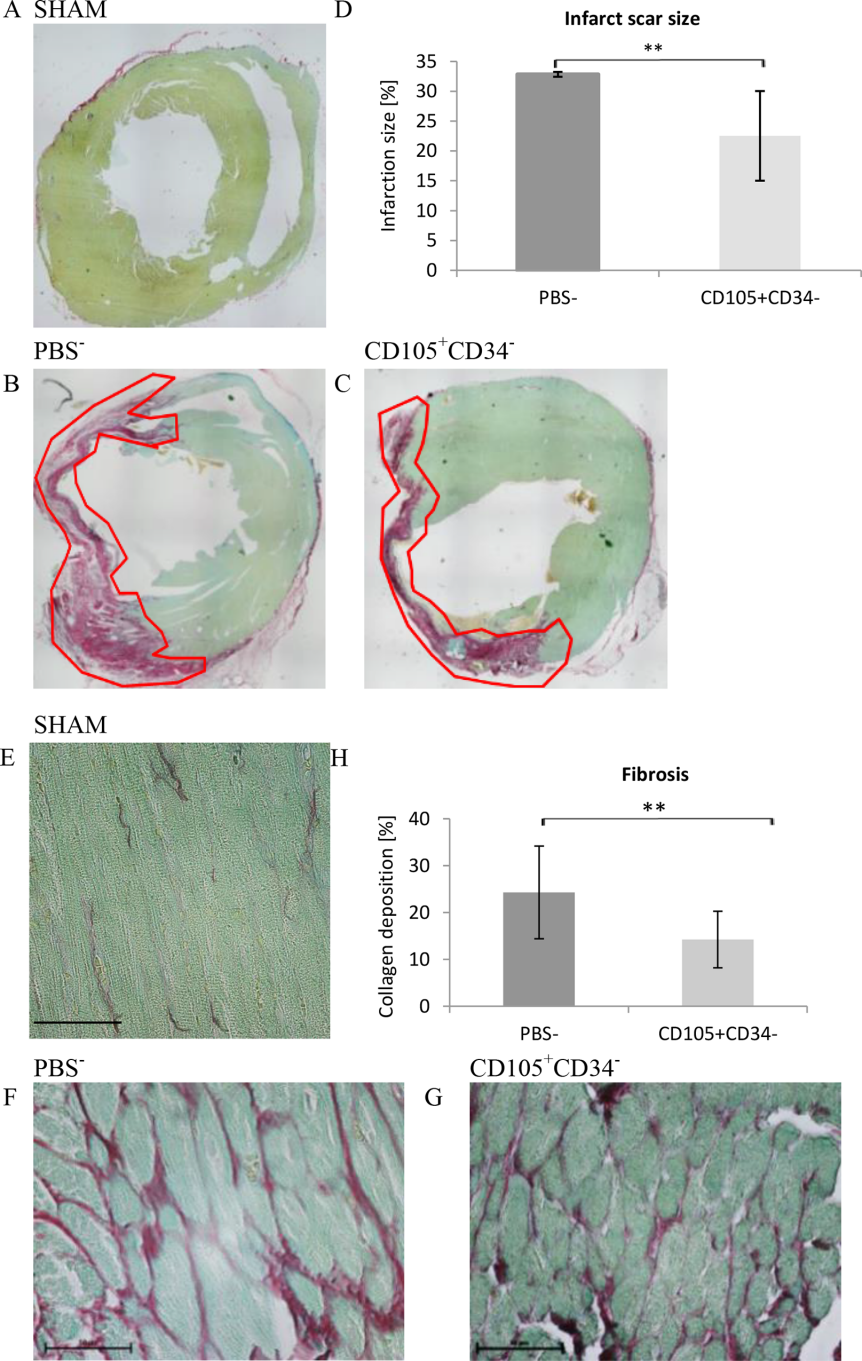
Changes in a size of the scar and fibrosis in the post-infarction heart of a mouse 7 weeks after MI. (A B C) Representative images illustrating a post-infarction scar in the mouse heart in a (A) Sham group or after administration of (B) PBS^-^ or (C) CD105^+^CD34^-^ cells. The images are composed of several shots (magn. 4x). (D) The area of the post-infarction scar is significantly smaller after administration of CD105^+^CD34^-^ cells compared to the control group, in which PBS^-^ was administered (n = 14); **p<0.01 by the U Mann–Whitney test. (E F G) Representative images illustrating collagen (pink) deposited between the fibers of the muscle tissue (green) in the mouse heart in a (E) Sham group or after administration of (F) PBS^-^, or (G) CD105^+^CD34^-^ cells. The sections with the greatest ratio of post-infarction scar to the whole stained area were used for examination (magn. 40x). (H) A statistically significant reduction of fibrosis was observed in the border area of the post-infarction scar after administration of CD105^+^CD34^-^ cells compared to the control group after administration of PBS^-^ (n = 14); **p<0.01 by the U Mann–Whitney test.

Fig 6A–6C presents representative images of histochemical staining of a post-infarction scar 6 weeks after administration of CD105^+^CD34^-^ cells or PBS^-^. In mice injected with CD105^+^CD34^-^ cells 7 days after MI, a statistically significant reduction in the size of post-infarction scar (22.53±5.55%) compared to the control PBS^-^ group (32.84±3.76%; p<0.01) was observed (Fig 6D).

The deposition of collagen between the muscle fibers in the border area of the post-infarction scar is associated with remodeling of the post-infarction heart. Fig 6E–6G shows representative images of collagen staining deposited between the cells of the muscle tissue in the border area of the post-infarction scar. In mice injected with CD105^+^CD34^-^ cells a statistically significant reduction in the amount of deposited collagen (14.23±3.02%), compared to the control PBS^-^ group (24.29±4.95%) (p<0.01) was observed (Fig 6H).

### 3.6. CD105^+^CD34^-^ cells improve the left ventricular ejection fraction after MI

**Fig 7:**
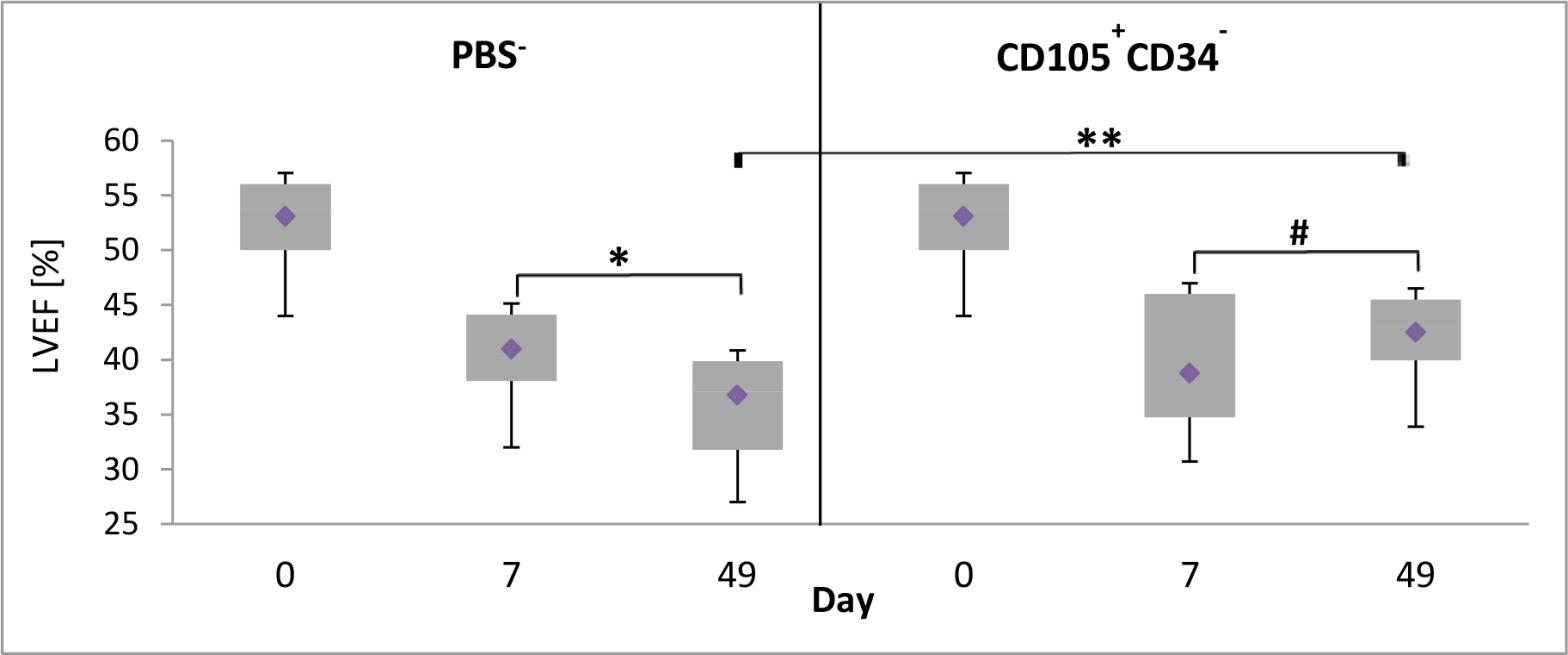
Changes in left ventricular ejection fraction (LVEF) 6 weeks after the cells administration. LVEF increased by 5.75% between treated groups at 49 day. Day 0 –LVEF (baseline); day 7 –LVEF seven days after MI induction, the day of administration of CD105^+^CD34^-^ cells or PBS^-^: day 49 –LVEF on the day of the collection of the hearts. PBS^-^: n = 16; CD105^+^CD34^-^: n = 18; **p<0.01; *p<0.05; # p = 0.0946. Comparisons between groups and within groups were performed by the one-way analysis of variance (ANOVA) with repeated measures with post-hoc Tukey tests.

The ultrasound examination of left ventricle was performed: before LAD ligation, 7 days after LAD ligation, before administration of the cells or PBS^-^, and 42 days after administration of CD105^+^CD34^-^ cells or PBS^-^. The measured parameter was left ventricular ejection fraction (LVEF). An improvement of functional parameters of infarcted heart was observed only in a group of mice injected with CD105^+^CD34^-^ cells. The myocardial contractility increased from 38.8% LVEF (after MI) to 42.5% (p = 0.0946). A statistically significant increase of LVEF was observed between a group of mice treated with CD105^+^CD34^-^ cells and a group of mice injected with PBS^-^ (36.8% vs 42.5%, respectively, p<0.01) (Fig 7).

### 3.7. IL-6 secretion after CD105^+^CD34^-^ cells administration and the presence of macrophages with M2 phenotype in ischemic mouse heart tissue

**Fig 8:**
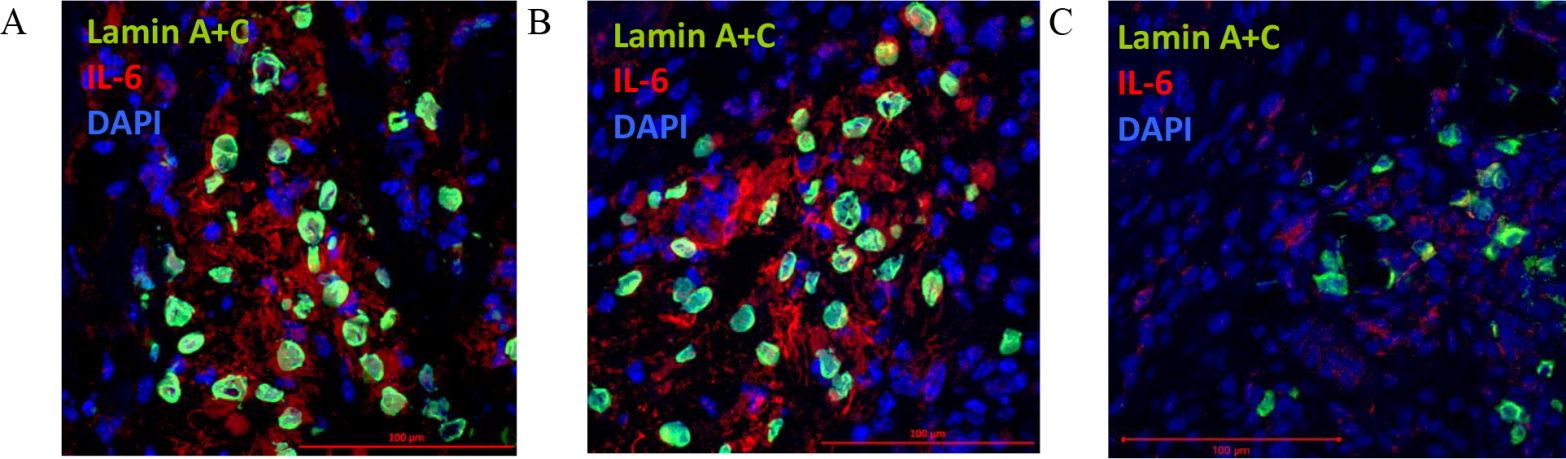
CD105^+^CD34^-^ cells secrete IL-6 after administration into the post-infarction mouse heart. CD105^+^CD34^-^ cells (lamin A+C, green) administrated into the border area of the post-infarction scar secreted IL-6 (IL-6, red) after 1 day (A), 3 days (B). After 7 days a trace amount of CD105^+^CD34^-^ cells were observed and IL-6 was slightly secreted (C) (magn. 40x).

**Fig 9:**
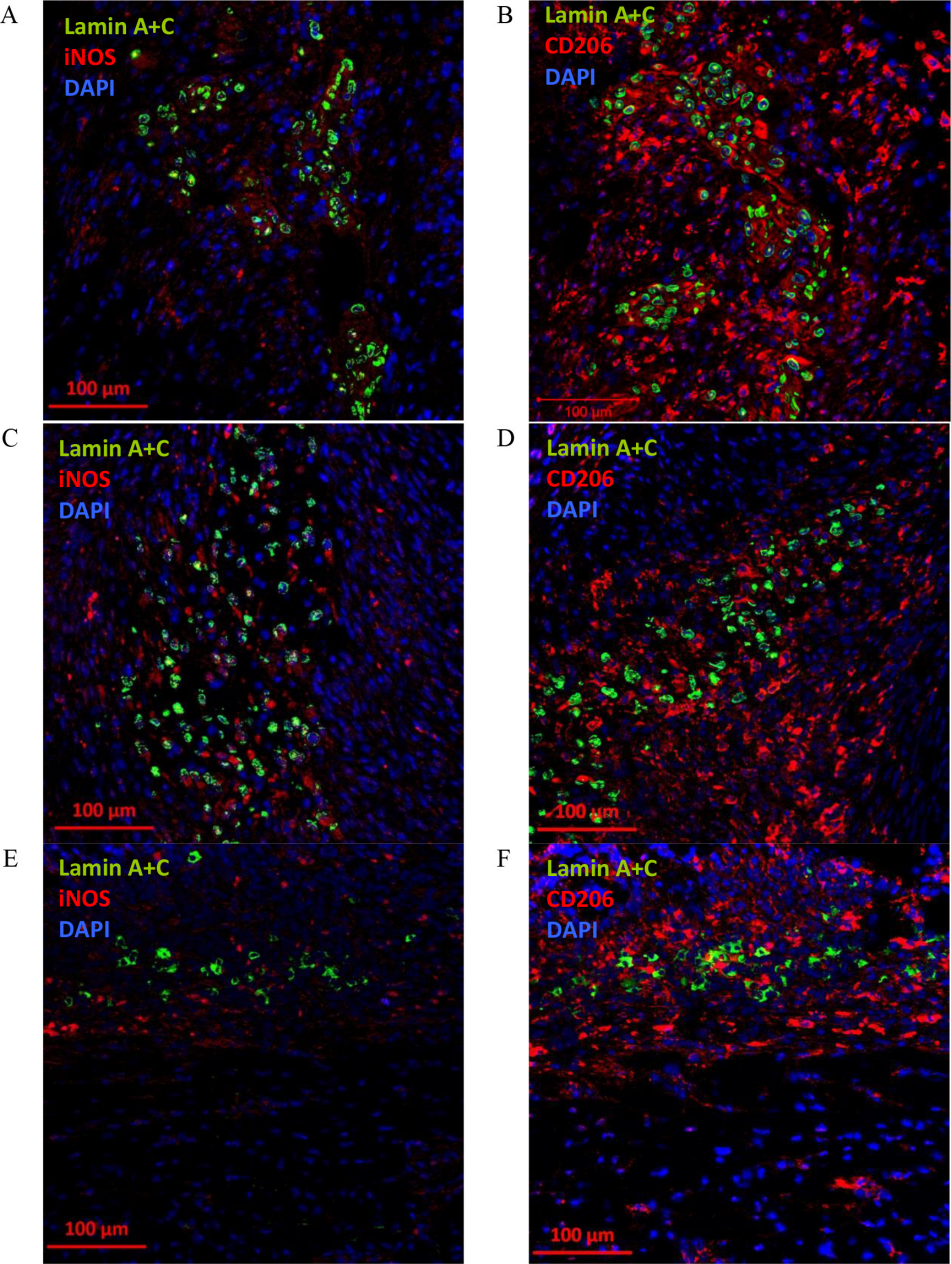
The presence of M2 macrophages after administration of CD105^+^CD34^-^ cells into the post-infarction mouse heart. (A) 1 day after implantation of CD105^+^CD34^-^ cells (lamin A+C, green) into the border area of the post-infarction scar, small amounts of pro-inflammatory macrophages with the M1 phenotype (iNOS, red) around the transplanted human cells were observed (magn. 20x). (B) 1 day after CD105^+^CD34^-^ cells (lamin A+C, green) implantation an increase in the number of anti-inflammatory, and proangiogenic macrophages with the M2 phenotype (CD206, red) in their surroundings was observed. The similar phenomenon was observed: (C) (D) 3 days after implantation of CD105^+^CD34^-^ cells (lamin A+C, green) and (E) (F) 7 days after implantation of CD105^+^CD34^-^ cells (lamin A+C, green) (magn. 20x).

When CD105^+^CD34^-^ cells were administrated into the border area of the post-infarction scar they retain their ability to IL-6 secretion (Fig 8). IL-6 is secreted at the first and third day after CD105^+^CD34^-^ cells administration (Fig 8A and 8B). The cytokine secretion is related with the cells presence in the tissue, and at the day seventh CD105^+^CD34^-^ cells and IL-6 were slightly detected (Fig 8C). Additionally, around the implanted human cells an increase in the number of anti-inflammatory, and proangiogenic macrophages with the M2 phenotype (CD206, C mannose receptor type 1) at all time points was observed (Fig 9). However, small amounts of pro-inflammatory macrophages with the M1 phenotype was also observed (iNOS, enzyme, inducible nitric oxide synthase). A trace amount of CD105^+^CD34^-^ cells were still observed in the tissue of post-infarcted heart up to 7 days after the cells administration (Fig 9E and 9F).

**Fig 10:**
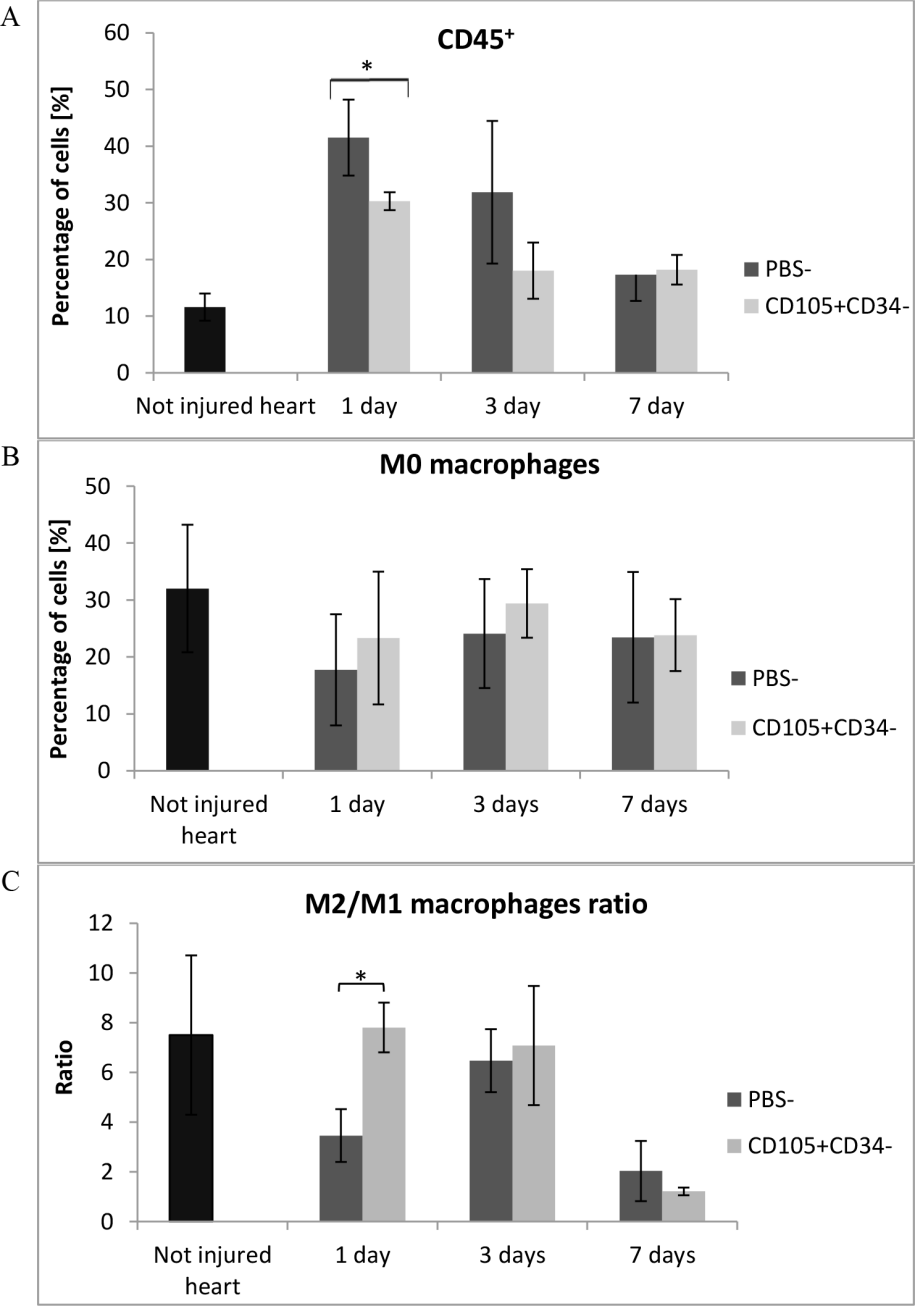
MSC with CD105^+^CD34^-^ phenotype modulate inflammation after administration into infarcted mouse heart. (A) The number of leukocytes (CD45^+^ cells) infiltrating the post-infarcted heart were significantly lower 1 day after CD105^+^CD34^-^ cells administration; n = 3. (B) The level of M0 macrophages (% of CD45^+^F480^+^ cells) accumulated in the infarcted heart was higher after CD105^+^CD34^-^ cells administration; n = 3. (C) CD105^+^CD34^-^ cells significantly increased the M2/M1 ratio 1 day after the cells administration; n = 3 *p<0.05 compared to the control (PBS^-^) group by the Student’s t-test.

To verify and compare the obtained results with the control group, the flow cytometry analysis of whole collagenase–digested mice hearts were performed. We characterized both cells of the immune system and cardiac macrophage subsets in the infarcted heart at the certain points after MI. Leukocytes were stained with anti-CD45 antibody and gated with CD45 fluorescence versus side scatter. The number of CD45^+^ cells infiltrating the post infarcted tissue was significantly lower (p<0.05) in hearts collected 1 day after CD105^+^CD34^-^ cells injections in comparison with the control group (Fig 10A). The number of CD45^+^ cells was over 3-fold lower (average: 1.8x10^6^ vs. 0.5x10^6^ cells). The level of macrophages M0 (CD45^+^F480^+^ cells) was higher in the in mice injected with CD105^+^CD34^-^ cells (Fig 10B). Two macrophage subsets were identified: M1 (CD45^+^F480^+^CD86^+^ cells) and M2 (CD45^+^F480^+^CD206^+^ cells). The percentage of M1 macrophages was relatively constant (< 5%) during first days after the cells or PBS^-^ injections and did not differ between groups (data not shown). However, the percentage of M2 macrophages was higher in mice injected with CD105^+^CD34^-^ cells 1 and 3 days after injections. Moreover, in those mice 1 day after the cells implantation the M2/M1 ratio was significantly higher compared to the control group (7.8 vs. 3.5) (p<0.05) (Fig 10C).

### 3.8. MSC-CM induced the expression of CD206 in BMDM

**Fig 11:**
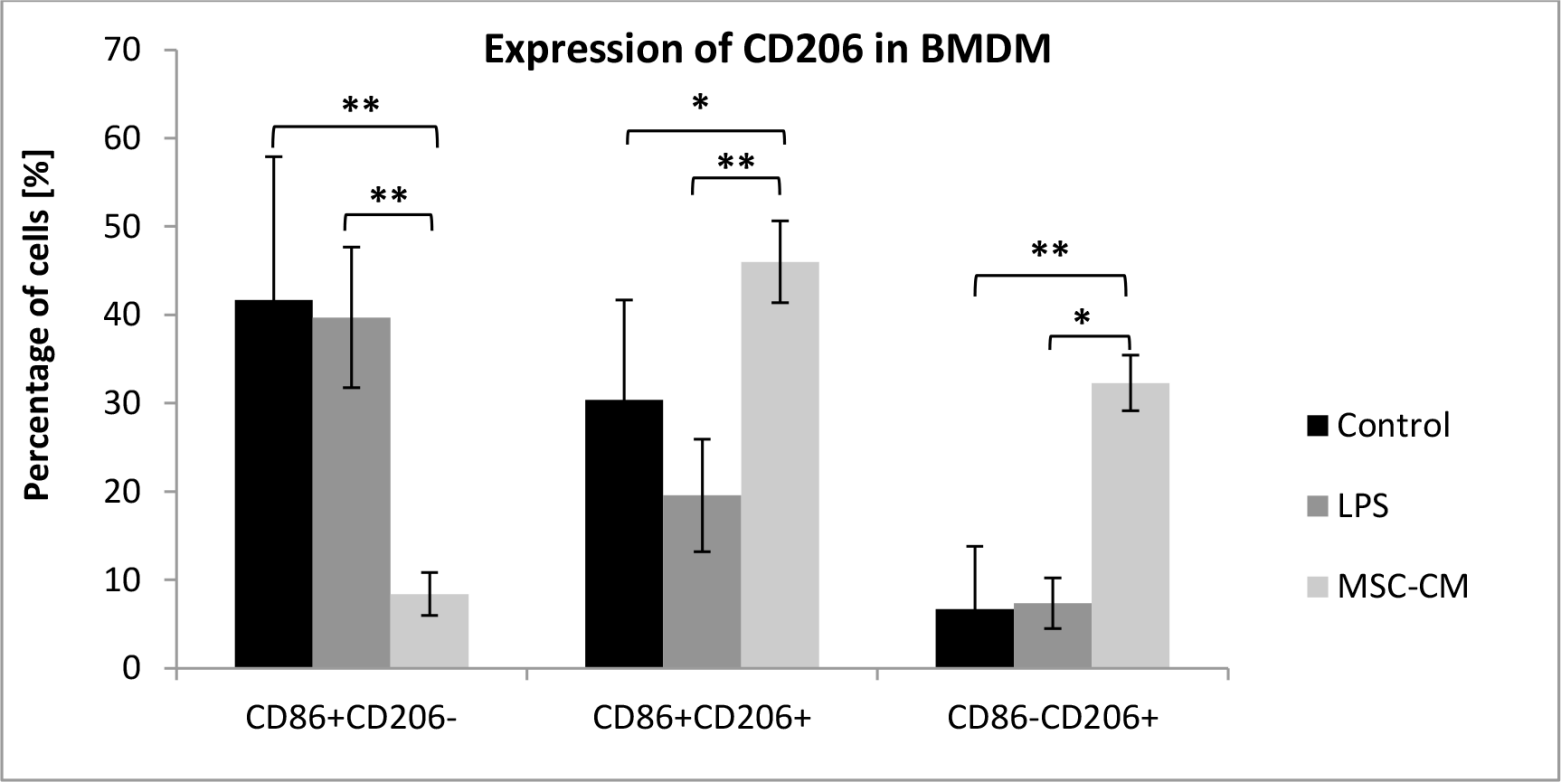
MSC-CM increased the expression of CD206 in BMDM. The incubation of MSC-CM with BMDM greatly increased the percentage of CD206^+^CD86^+^ and CD206^+^CD86^-^ macrophages in comparison with control BMDM cells and BMDM cells incubated in medium with LPS; n = 6 *p<0.05; **p<0.01 by one-way analysis of variance (ANOVA), using Tukeys’ multiple comparison test for post hoc analysis for the groups: CD86^+^CD206^-^ and CD86^+^CD206^+^; by Kruskal–Wallis one-way analysis of variance, using multiple comparison of mean ranks for all groups, for CD86^-^CD206^+^ group.

To directly assess if CD105^+^CD34^-^ cells induce an M2 phenotype in macrophages we determined the expression of mannose receptor (CD206), a well-accepted marker for M2 macrophages, in BMDM using flow cytometry. The incubation of MSC-CM with BMDM greatly increased the percentage of CD206^+^CD86^+^ and CD206^+^CD86^-^ macrophages (46% and 32.3%, respectively) than in the control BMDM (30.4% and 6.7%, respectively) and in BMDM incubated in medium with LPS (19.6% and 7.4%, respectively) (Fig 11). All analyzed BMDM expressed F4/80 antigen, a characteristic marker for macrophages (data not shown).

## 4. Discussion

The aim of the present study was to investigate therapeutic potential of human MSC with CD105^+^CD34^-^ phenotype in a mouse model of hindlimb ischemia and myocardial infarction. We aimed also to investigate whether MSC may be transplanted into fully immunocompetent recipients without immunosuppression. The unique immune privileged nature of MSC may prevent rapid rejection in a xenogeneic environment and preserve their ability to promote the repair of injured tissues [30,31].

Population of cardiac cells with CD105^+^CD34^-^ phenotype was selected with the use of FACS (Fluorescence-Activated Cell Sorting) thereby were characterized by specific immunophenotype. Theoretically, the cells population should exhibit reduced heterogeneity. The population of these cells, however, expresses all antigens characteristic for MSC and differentiate *in vitro* into adipocytes, chondroblast and osteoblasts (Fig 1).

CD105^+^CD34^-^ cells in the culture conditions secrete mainly: IL-6, IL-8, GRO, OPG and HGF. However, the predominant cytokine is IL-6 (Fig 2). Some papers suggest that IL-6 may induce cardiomyocytes hypertrophy, fibroblasts proliferation and elevated fibrosis, contributing to adverse myocardial remodeling [32, 33]. IL-6 participates also in immune reactions. Depending on the microenvironment the cytokine may be pro- or anti-inflammatory one [34]. IL-6 is involved in polarization of M1 macrophages towards macrophages with M2 phenotype [35,36]. M1 are pro-inflammatory macrophages. M1 secrete a toxic effector molecules (ROS and NO) and pro-inflammatory cytokines (IL-1β, TNF, IL-6) [37]. M2 are anti-inflammatory macrophages. M2 secrete anti-inflammatory cytokines mainly IL-10 and TGFβ [36]. OPG cytokine (a member of the tumour necrosis factor receptor (TNFR) superfamily) stimulates the secretion of IL-6 cytokine [38]. IL-8 may act as pro-inflammatory cytokine able to recruit monocytes and neutrophils. In addition to the pro–inflammatory cells recruitment, IL-8 serves to promote their activation [39, 40]. On the other hand, IL-8 together with GRO, and HGF cytokines are associated with angiogenesis promotion. Therefore, the predominant cytokines secreted by cells with CD105^+^CD34^-^ phenotype are cytokines that stimulate the formation of M2 phenotype in macrophages and cytokines with proangiogenic properties [41]. We have shown, both in a mouse model of hindlimb ischemia and myocardial infarction, that MSC with CD105^+^CD34^-^ phenotype exhibit therapeutic properties. MSC facilitated fast functional recovery of ischemic limb and promoted angiogenesis (Fig 3). In the infarcted hearts MSC stimulated: (1) an increase of vascularity, both in the border area of the post-infarction scar, and within the scar (Figs 4 and 5), (2) a reduction of the post-infarction scar and fibrosis (Fig 6), (3) an increase of the left ventricular ejection fraction (Fig 7).

How do human MSC exert their beneficial effect? MSC are short-lived cells. After intravenous infusion they are present in the lungs up to 24 hours [42]. MSC retention time in the heart is also short: 4 hours after intramyocardial administration there is 10% of injected MSC, 24 hours later only 1% [43]. In our experiments trace amount of CD105^+^CD34^-^ cells were still observed 7 days after the cells injections directly into infarcted heart (Fig 8). Should we consider this relatively short retention time as sufficient to enable transplanted cells to transdifferentiate and to replace the damaged cells? It appears, that this time is adequate to stimulate an activity of other cells via MSC secreted factors (so-called “cell empowerment” described by Wang et al.[10]). The cells which are stimulated by MSC are mainly macrophages [28,44,45].

The primary mechanism of MSC action may be described as "hit and run" (see: Ankrum et al. [46]). Short-lived MSC secrete a various paracrine factors: cytokines and growth factors, which, among others, inhibit apoptosis, fibrosis, activity of immune cells, induce angiogenesis [43]. Among these factors there is also IL-6. IL-6 participates in polarization of M1 macrophages towards M2 phenotype [35,36]. In the M1→M2 macrophage polarization may be involved also other growth factors (e.g. VEGF, TGF-β, PLGF, GM-CSF), chemokines (CCL2) or interleukins (e.g. IL-4, IL-10, IL-21) [47].

Our study supports the hypothesis of macrophages "educated" by MSC [44]. CD105^+^CD34^-^ cells in the ischemic mouse heart tissue secreted IL-6, a cytokine known for macrophages polarization (Fig 8). In the area of post-infarction scar, where CD105^+^CD34^-^ cells were transplanted, we observed the influx of M2 macrophages (cells with F4/80^+^CD206^+^ phenotype) (Figs 9 and 10C). Furthermore, in the culture of murine macrophages stimulated with human MSC (CD105^+^CD34^-^) conditioned medium, we noted an increase in the level of CD206 (a marker of M2 macrophages) (Fig 11). *In vitro* CD105^+^CD34^-^ cells polarized macrophages towards M2 phenotype. We believe that secreted IL-6 may be the cytokine that contributes to M1→M2 polarization. M2 macrophages may be, in fact, the therapeutic cells. M2 cells stimulate, inter alia, angiogenesis [48].

The therapeutic effect, which was achieved in our work, differs in some details from the therapeutic effect obtained by Rossini et al. [19]. According Rossini et al. [19] MSC derived from the human heart, so-called cardiac stromal cells (CStC), when injected into the rat post-infarcted heart, persisted longer within the tissue than bone marrow mesenchymal stromal cells (BMStC). CStC migrated into the scar, differentiated into adult cardiomyocytes more effectively than BMStC. CStC contributed to new blood vessels formation. The secretome analysis showed that both IL-6 and LIF (Leukemia inhibitory factor) were increased in CStC-conditioned media. Our data support Rossini et al. [19] results, that CStC contribute to an increase in the number of blood vessels in the ischemic area, as well as secrete IL-6 cytokine. Nevertheless, the transdifferentiation of CStC into the cardiomyocytes is a controversial event, rarely observed *in vivo* [49].

On the other hand Gaebel et al. [16] compared the therapeutic potential of human MSC (hMSC) derived from different sources (umbilical cord blood, adipose tissue, bone marrow). All isolated hMSC populations showed to a certain extent a therapeutic potential. Nevertheless, CD105^+^ revealed overall a better myocardial performance. The intracardiac CD105^+^ cells injection showed a significant functional improvement of left ventricle. The pure fraction of CD105^+^ hMSC exhibited a favorable survival pattern of infarcted hearts which translated into a more robust preservation of cardiac formation [16].

Do xeno- or allografts may be recognized and rejected by host immune system? The success of therapy involving allografts indicates that it is unlikely (see: Ankrum et al. [42]). This doubt was attempted to explain by MSC immune privilege phenomenon [49]. MSC lack MHC class II molecules, express low levels of MHC class I, the cells lack the expression of costimulatory molecules CD80 and CD86. Hence, they are thought to be not recognized by the host immune system [50]. Nevertheless, under appropriate conditions, allogeneic MSC induce a memory T-cell response resulting in rejection of an allogeneic donor graft [51]. Also IL-2-activated NK cells effectively lyse autologous and allogeneic MSC [52].

In our study, both in the mouse model of hindlimb ischemia and myocardial infarction, we did not notice, however, rejection of transplanted human CD105^+^CD34^-^ cells by the host organism. It may be that short-lived MSC are not able to induce an immune response. The number of mononuclear cells (MNC: lymphocytes, monocytes, and dendritic cells) isolated from infracted hearts was almost 3-times lower in mice which human CD105^+^CD34^-^ cells were administrated compared to the control group (data not shown). And even if such a response appears, it is worth remembering that M2 macrophages are presumably the effector (therapeutic) cells, not MSC. We believe that allogeneic and xenogeneic grafts can be used to test on the properties of MSC and their sub-populations. Especially in therapeutic models with the use of small laboratory animals.

In summary, we have shown that the examined cells (xenogeneic transplants) have therapeutic properties: they inhibit the inflammatory response, reduce fibrosis and infarct size, increase vascularization in the ischemic hindlimb and post-infarction heart, and increase left ventricular ejection fraction.

## Supporting Information

S1 Text A detailed description of the methodology. (DOCX)
