# Supplementary material for: Human Cardiac Mesenchymal Stromal Cells with CD105+CD34- Phenotype Enhance the Function of Post-Infarction Heart in Mice
Source: PLoS One. 2016 Jul 14;11(7):e0158745. doi: 10.1371/journal.pone.0158745 (PMC4945149; doi:10.1371/journal.pone.0158745)
Supplement: S1 Text — (DOCX) [file pone.0158745.s001.docx]

# Supplementary material

## S1. A detailed description of the methodology

## Biological material used in the study

The material comprised the hearts of recipients (n=19), excised during transplant surgeries in the Silesian Centre for Heart Diseases in Zabrze (Poland). The patients underwent heart transplantation surgery owing to extreme heart failures of different etiologies. Fragments of tissue collected from the right ventricle were used to isolate cells.

## Isolation of MSC

The tissue collected from the hearts was minced into fragments of approximately 1mm^3^ and digested with collagenase NB4 solution (Serva Electrophoresis, Heidelberg, Germany) for 4h at 37ºC [19]. After the digestion, the cell suspension was filtered through 70µm and 40µm strainers. The suspension of single cells was placed in a plastic culture plates in IMDM medium (Sigma Aldrich, St Louis, MO, USA), supplemented with 20% FBS (Gibco BRL, Paisley, UK), L-glutamine (Sigma Aldrich), basic fibroblast growth factor (bFGF, 10ng/ml, eBioscience, San Diego, CA, USA), and antibiotics (Sigma Aldrich). After 48 hours, the plates were rinsed with a fresh culture medium.

## Isolation of CD105^+^CD34^-^ cells

After obtaining 90-100% confluence of MSC, the cells were treated with 0.25% trypsin, and subsequently incubated for 30min. at 4ºC with antibodies directed against the following human antigens: CD105 – APC and CD34 – PE-Cy7 (BD Pharmingen), or isotype-matched control antibodies. The cell suspension was incubated with DAPI (0.2µg/ml, 10min., Sigma Aldrich). The population of CD105^+^CD34^-^ cells was separated using a BD FACSAria™ III cell sorter (BD Biosciences). CD105^+^CD34^-^ cells were cultured under the same conditions as the freshly isolated MSC.

## Differentiation of MSC with CD105^+^CD34^-^ phenotype

MSC were cultured for 21 days in the following differentiation media: Adipogenic Differentiation Medium, Osteogenic Differentiation Medium, and Chondrogenic Differentiation Medium. The ability of CD105^+^CD34^-^ cells to differentiate into adipocytes was assessed by immunofluorescence staining, using an antibody directed against the FABP4 (Fatty Acid Binding Protein, Abcam, Cambrige, UK), and a secondary antibody linked to an FITC fluorochrome (Fluorescent anti-rabbit IgG Kit, Vector Laboratories, Burlingame, CA, USA). Fluorescence imaging of the stained cells was performed using a LSM710 confocal microscope (Carl Zeiss Microscopy GmbH). The ability of the cells to differentiate into chondroblasts and osteoblasts was assessed by histochemical staining using Safranin O and Alizarin Red (Sigma, Aldrich, respectively). The stained cells were visualized using an Eclipse 80i microscope (Nikon Instruments Inc., Melville, NY, USA).

## Analysis of the secretome of CD105^+^CD34^-^ cells

CD105^+^CD34^-^ cells (1^st^ passage) were plated at high density in a complete medium. On the next day of the culture, the medium was replaced with an IMDM without serum. After 4h the medium was replaced with a fresh one. After 48h the medium was collected. A sample of the medium was incubated overnight at 4ºC in a chamber with a membrane coated with antibodies directed against the examined cytokines. The membrane was subsequently incubated with a kit of biotinylated secondary antibodies, HRP-labeled streptavidin, and a chemiluminescent substrate for horseradish peroxidase. A chemiluminescent emission was detected using G-BOX device (Syngene, Cambrige, UK). Densitometry calculations were performed using an Image J 1.48y software to quantity analyze of cytokines and growth factors secreted by CD105^+^CD34^-^ cells.

## Mouse model of hindlimb ischemia

Unilateral femoral artery ligation was performed according to Brenes et al. [23] on male C57Bl/6NCrl mice (8-10-week-old). Animals were anesthetized with 2% isoflurane (MiniVent Model 845, Harvard Apparatus, USA). Skin from the inner side of the left hindlimb were incised over a length of about 1cm and the superficial femoral artery were exposed. The artery was ligated at two points using surgical sutures. First ligation was performed about 0.5cm from the hip joint and the other about 1cm further down from the first ligation. An hour after ligation, 10^6^ CD105^+^CD34^-^ cells in 100μL of PBS^-^ were administered into the muscle. The control mice were injected with 100μL of PBS^-^. After 14 days mice were sacrificed and the muscles were collected for further immunohistochemical analysis

## Induction of myocardial infarction (MI) and CD105^+^CD34^-^ cells implantation

MI was induced in 8-10-week-old C57Bl/6NCrl male mice. After intubation, mice were anesthetized with 2% isoflurane (MiniVent Model 845). The branch of the left anterior descending coronary artery (LAD) was permanently ligated with a monofilament suture, Prolene 8-0, accessed through a left anterior thoracotomy. Ischemia was confirmed by paling of the wall of the left ventricle, distally to the completed suture. 7 days after LAD ligation the mice were subjected to reoperation. 0.5x10^6^ CD105^+^CD34^-^ cells in 10µl of PBS^-^ (4 injections in a volume of 2.5µl each) or 10µl of PBS^-^ (4x2.5 µl) (control), were injected into the border area of the post-infarction scar, using a syringe and needle for microinjections (Hamilton, USA). The sham group consisted of mice injected with PBS^-^ without LAD ligation. Hearts were collected 42 days after PBS^-^ or CD105^+^CD34^-^ cells injection [16].

## Echocardiographic evaluation

Echocardiographic examinations were conducted using Vevo®2100 Imaging System (VisualSonics, Toronto, Canada), equipped with a MS400 head with a resolution of 300MHz. The ultrasound examination was performed: before LAD ligation, 7 days after LAD ligation, before administration of the cells/PBS^-^, and 42 days after administration of CD105^+^CD34^-^ cells or PBS^-^. The animals were anesthetized with 2% isoflurane (MiniVent Model 845). The concentration of isoflurane on the test platform equipped with ECG electrodes was adjusted so as to obtain a sufficient depth of sedation (HR 400-500). The heart was examined from the parasternal long axis view (PSLA) with a simultaneous monitoring of physiological parameters (ECG and respiration rate). The analysis of the left ventricular ejection fraction (LVEF) was performed using the LV-Trace volume package of the Vevo®2100 system.

## Preparation of the tissue for immunohistological analyses

The collected muscles and hearts were fixed with 4% paraformaldehyde, and then embedded in paraffin or frozen in cooled isopentane (2-methylbutane, Sigma Aldrich) and liquid nitrogen. Subsequently, tissues were sectioned into 5-8µm slices.

## Evaluation of the size of the post-infarction scar and its state of fibrosis

The paraffin sections were incubated with a 0.1% solution of Fast Green and a 0.1% solution of Sirius Red (Sigma) in 1.2% picric acid (1h, RT). The sections with the greatest ratio of post-infarction scar to the whole stained area were used for immunohistochemical examination. The size of the post-infarction scar was presented as the percentage ratio of the areas stained pink (collagen) to the sum of areas stained green and pink (muscle tissue + collagen). The fibrosis was evaluated as a ratio of collagen deposited between the muscle fibers in 5 randomly selected fields in the border area of the post-infarction scar. Microscopic observations were performed using a Nikon Eclipse 80i microscope. The percentage share of the stained areas of tissue was determined using the NIS Elements AR 3.1 software.

## Determination of the number of blood vessels

Lectin (*Lycopersicon Esculentum* Lectin LEL, Vector Laboratories) conjugated with FITC was used to determine the number of blood vessels in the border area of the post-infarction scar. Paraffin sections were incubated with lectin. Blood vessels were counted in 10 randomly selected fields, in the border area of the post-infarction scar. In order to determine the number of blood vessels in the area of the post-infarction scar, the paraffin sections were incubated with anti-CD31 antibody (Abcam) following 2.5% goat serum (Vector Laboratories) incubation and subsequently with a secondary antibody conjugated with FITC (Vector Laboratories). Sections were mounted in VECTASHIELD Mounting Medium with DAPI (Vector Laboratories). Stained vessels were counted in 5-8 randomly chosen fields. Also the number of blood vessels were determined in the hindlimb ischemic muscles. Frozen sections were incubated with an antibody directed against CD31 antigen (Abcam) and subsequently with a secondary antibody conjugated with FITC (Vector Laboratories). Sections were mounted in VECTASHIELD Mounting Medium with DAPI (Vector Laboratories). Stained vessels were counted in 10 randomly chosen fields per section. Blood vessels were counted in 10 muscles of each group. The result was expressed as number of blood vessels per 1mm^2^. Microscopic observations were performed using a LSM710 confocal microscope (Carl Zeiss Microscopy GmbH).

## Identification of M1 and M2 macrophages

After CD105^+^CD34^-^ cells transplantation into the border area of the post-infarction scar, the hearts were collected at the following time points: 1, 3 and 7 days. Frozen sections were incubated with an antibody directed against CD206 antigen (Abcam) or iNOS enzyme (Abcam), and subsequently with a secondary antibody conjugated with Texas Red (Vector Laboratories). Human CD105^+^CD34^-^ cells were identified in a mouse heart by an antibody directed against human lamin A+C, Nuclear Envelope Marker (Abcam). Sections were immunostained following the instructions for the Vector M.O.M. Fluorescein Kit (Vector Laboratories).

Flow cytometry was used to determine macrophages subset in the post-infarcted hearts. At the following time points: 1, 3 and 7 days, hearts were collected, minced and digested with collagenase II solution (Gibco; 500U/mL) for 1h at 37ºC. The isolated cells were blocked with anti-mouse CD16/32 antibody (10min.; eBioscience) and then incubated for 30min. at 4ºC with antibodies directed against the following mouse antigens: CD45 – PE-Cy7, F4/80 – PE (eBioscience), CD86 – APC (as M1 marker; BD Pharmingen), CD206 – FITC (as M2 marker; Serotec a Bio-Rad Company, Oxford, UK) or isotype-matched control antibodies. Macrophages phenotype was determined using a flow cytometer (BD FACSCanto ™ BD Biosciences). Dead cells were eliminated by using the viability dye 7AAD (BD Pharmingen). Side scatter and forward scatter profiles were used to eliminate cell doublets.

## Identification of IL-6 in the mouse tissues

After CD105^+^CD34^-^ cells transplantation into the border area of the post-infarction scar, the hearts were collected at the following time points: 1, 3 and 7 days. Frozen sections were incubated with an antibody directed against IL-6 (Abcam), and subsequently with a secondary antibody conjugated with Texas Red (Vector Laboratories). Human CD105^+^CD34^-^ cells were identified in a mouse heart by an antibody directed against human lamin A+C, Nuclear Envelope Marker (Abcam). Sections were immunostained following the instructions for the Vector M.O.M. Fluorescein Kit (Vector Laboratories).

## The isolation of mouse BMDM (Bone Marrow-Derived Macrophages)

BMDM were isolated from 8-10-week-old C57Bl/6NCrl male mice. The femurs and tibias were separated and the bones were flushed with RPMI-1640 medium containing 10% fetal bovine serum [25]. Bone marrow-derived cells were cultured for 7 days in macrophage differentiation media (30% L929 cell-conditioned medium, 20% fetal bovine serum and 50% RPMI-1640). L929 cell-conditioned medium was prepared by growing L929 (ATCC, Manassas, USA) cells in RPMI-1640-containing 10% FBS for 10 days. The medium containing macrophage colony-stimulating factor secreted by the L929 cells was harvested and passed through a 0,22µm filter [26].

## Conditioned medium from CD105^+^CD34^-^ cells

After reaching confluence, CD105^+^CD34^-^cells were cultured in IMDM medium containing 20% FBS, L-glutamine and antibiotics for 48h to obtain conditioned medium (MSC-CM). The supernatants were collected and were stored at -20°C until use.

## Immunophenotypic analysis of BMDM

After BMDM incubation with appropriate medium the phenotype of cells were analyzed using flow cytometry (BD FACSCanto™, BD Biosciences). The cells were blocked for 15 min. with anti-mouse CD16/32 antibody (eBioscience) and then incubated for 30min. at 4ºC with antibodies directed against the following mouse antigens: CD206 – FITC (Serotec a Bio-Rad Company), CD86 – APC (BD Pharmingen) and F4/80 – PE (eBioscience) or isotype-matched control antibodies. Subsequently the cells were incubated with 7AAD (BD Pharmingen) (10min. RT).
